# Active surveillance of small renal masses

**DOI:** 10.1186/s13244-020-00853-y

**Published:** 2020-05-05

**Authors:** Carmen Sebastià, Daniel Corominas, Mireia Musquera, Blanca Paño, Tarek Ajami, Carlos Nicolau

**Affiliations:** 1 Radiology Department, CDIC, Hospital Clínic de Barcelona, C/Villaroel no. 170, 08036 Barcelona, Spain; 2 Urology Department, ICNU, Hospital Clínic de Barcelona, C/Villaroel no. 170, 08036 Barcelona, Spain

**Keywords:** Small renal mass, Renal cell carcinoma, Elderly, Diagnostic imaging, Delayed intervention

## Abstract

Most renal masses incidentally detected by cross-sectional images are benign, being mainly cysts, and if they are malignant, they are indolent in nature with limited metastatic potential. Enhanced renal masses less than 4 cm in size are known as small renal masses (SRMs), and their growth rate (GR) and the possibility of developing metastasis are extremely low. Delayed intervention of SRMs by closed and routine imaging follow-up known as active surveillance (AS) is now an option according to urological guidelines. Radiologists have a key position in AS management of SRMs even unifocal and multifocal (sporadic or associated with genetic syndromes) and also in the follow-up of complex renal cysts by Bosniak cyst classification system. Radiologists play a key role in the AS of both unifocal and multifocal (sporadic or associated with genetic syndromes) SRMs as well as in the follow-up of complex renal cysts using the Bosniak cyst classification system. Indeed, radiologists must determine which patients with SRMs or complex renal cysts can be included in AS, establish the follow-up radiological test algorithm to be used in different scenarios, perform measurements in follow-up tests, and decide when AS should be discontinued. The purpose of this article is to review the indications and management of AS in SRMs, especially focused on specific scenarios, such as complex renal cysts and multifocal renal tumors (sporadic or hereditary). In this work, the authors aimed to provide a thorough review of imaging in the context of active surveillance of renal masses.

## Key points

- Small renal masses are contrast-enhancing renal tumors less than or equal to 4 cm.
- Active surveillance is defined as initial management including the monitoring of renal tumor size.
- The follow-up protocol most commonly used includes enhanced abdominopelvic CT scanning.
- Progression is defined as a linear growth rate greater than 0.5 cm per year, diameter greater than 4 cm, or metastasis.

## Background

**Fig. 1:**
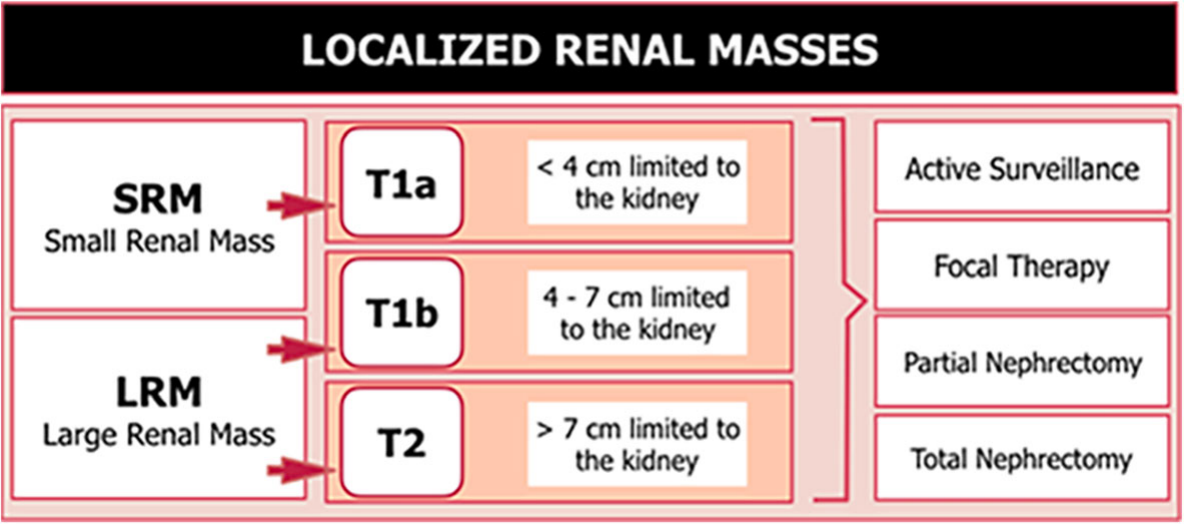
Definition, classification, and therapeutic approaches of localized renal masses. Localized renal masses were considered to be all the masses limited to the kidney which means no perinephric or renal sinus fat invasion. T1a, T1b, and T2 correspond to the TNM classification

**Fig. 2:**
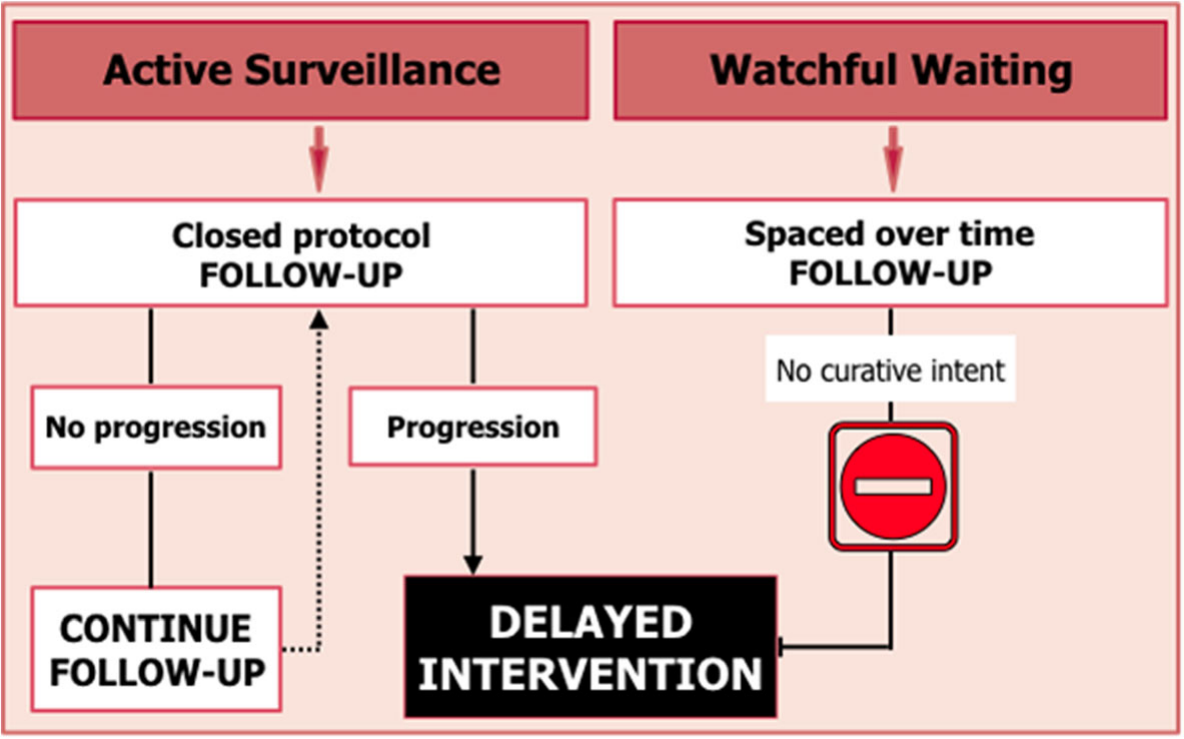
The main differences between active surveillance (AS) and watchful waiting (WW) in the monitoring of small renal masses. Active surveillance is defined as the initial management including the monitoring of renal tumor size by serial imaging with delayed treatment in case of progression. In WW, close monitoring with imaging techniques is not routine, curative treatment is not administered, and active treatment is only indicated on the appearance of symptoms

Over the last 20 years, the incidence of renal cell carcinoma (RCC) has increased globally due to advances in cross-sectional imaging of small renal masses (SRMs). SRMs are defined as incidentally image-detected, contrast-enhancing renal tumors less than or equal to 4 cm in diameter which are usually consistent with stage T1a renal cell carcinoma [1]. Adequate clinical management of SRMs is important despite their slow growth and rare metastatic potential. Optimal management of SRMs should balance the need for treatment, as around 25% of all small masses are benign lesions, with the goal of preserving renal function as far as possible and avoiding the risk of overtreatment [2]. An important proportion of SRMs are diagnosed in older patients or patients with multiple comorbidities, in whom surgery might outweigh the potential clinical benefits of active surveillance (AS). Active surveillance is defined as the initial management including the monitoring of renal tumor size by serial imaging with delayed treatment in case of progression and is now considered as an option in the treatment of localized renal tumors [3] (Fig. 1). Nonetheless, AS should be differentiated from the watchful waiting (WW) approach in which patient monitoring with imaging techniques is not routine, curative treatment is not administered and active treatment is not indicated unless symptoms appear [4] (Fig. 2).

The aim of this review is to describe the indications and practical management of AS in SRMs, especially focused on specific scenarios such as complicated renal cysts and multifocal renal tumors (sporadic or hereditary).

## Renal masses: indications for active surveillance

Small renal masses are the ones most indicated to be included in AS. Since SRMs tend to be indolent, with slow growth rates (1–3 mm per year), and are associated with a relatively low risk of metastasis (1–3%). AS is the most adequate treatment approach [5–7]. Large renal masses (LRM), which are defined as solid localized renal masses greater than 4 cm in size usually corresponding to T1b and T2 RCC, can also be followed by AS, although the more rapid growth (4–8 mm per year) and higher risk of M1 metastasis (4–6%) require that this approach be made with caution [8].

In the absence of level I evidence, the criteria for patients with SRMs to be eligible to receive AS are difficult to define. In various urologic guidelines, AS is considered to be a treatment option in elderly patients (although none defines a specific cutoff age), as well as in patients with multiple comorbidities and a limited life expectancy or with high perioperative morbid-mortality risk [2, 4, 9–11]. AS is also considered in chronic kidney disease or individual kidney patients in whom an observation period can be considered [12]. Only the recent American Urology Association (AUA) and the American Society of Clinical Oncology (ASCO) guidelines for SRMs provide an in-depth description of the indications of AS [2–4].

According to the 2017 AUA guidelines [4]:

- AS is an option for initial management in patients with renal masses suspicious for cancer, especially those smaller than 2 cm.
- AS or expectant management should be a priority when the anticipated risk of intervention or competing risks of death outweigh the potential oncologic benefits of active treatment.
- When the results of a risk-versus-benefit analysis of the treatment are equivocal and the patient elects to undergo AS.
- When the oncologic benefits of intervention outweigh the risks of treatment and competing risks of death, physicians should recommend active treatment. In this setting AS with potential for delayed intervention may be pursued only if the patient understands and is willing to accept the associated oncologic risk.

The ASCO guidelines define the indications of AS as follows [2]:

- Absolute indications: high risk for anesthesia and intervention or life expectancy less than 5 years
- Relative indications: significant risk of end-stage renal disease (ESRD) if treated, SRM less than 1 cm, or life expectancy less than 10 years

In clinical practice, the indication of AS is evaluated individually by tumor boards, with the risk of RCC treatment being balanced with the oncologic results of AS, patient age, comorbidities, and limited life expectancy, and finally, leading to multidisciplinary consensus.

## Renal masses: contraindications for active surveillance

AS should not be carried out in the following:

- Benign renal masses reliably diagnosed by imaging or biopsy
- Renal masses with irregular borders
- Non-localized renal tumors (i.e., locally, lymphatically, or hematogenously disseminated)
- When the oncologic benefits of intervention outweigh the risks of treatment and competing risks of death
- When the patient refuses to be included in AS

**Fig. 3:**
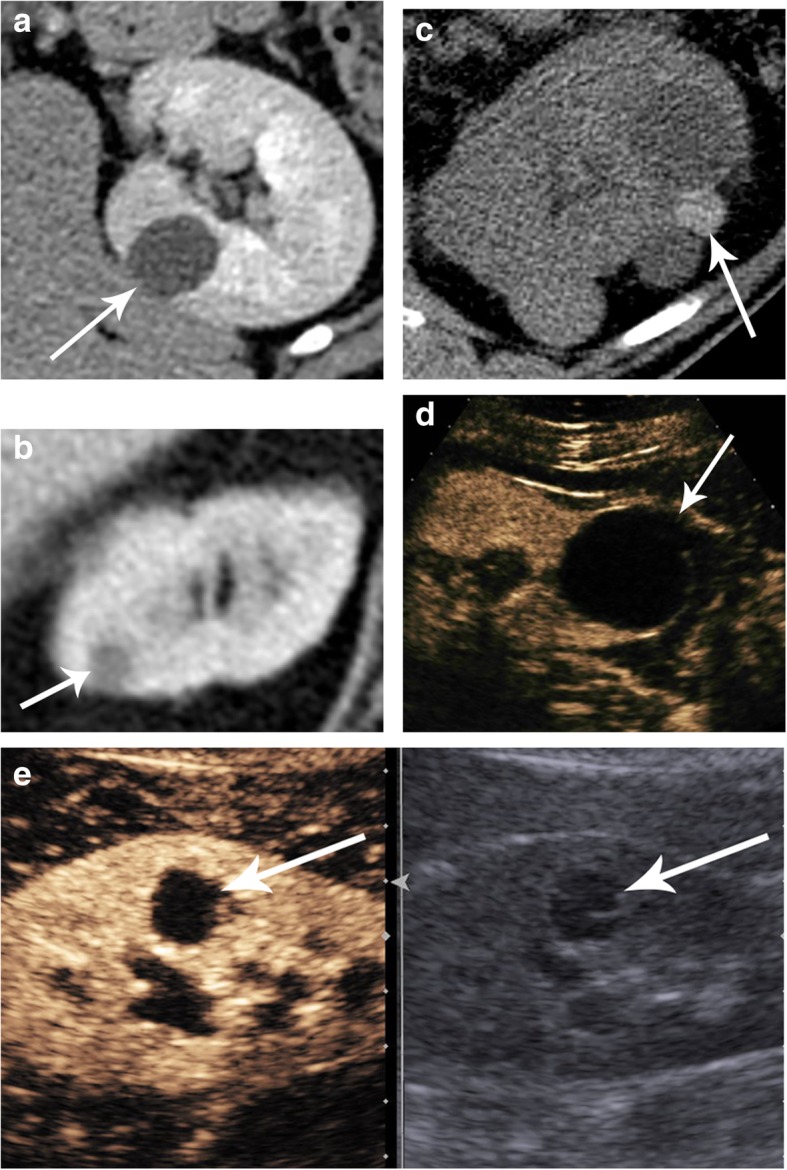
Renal masses that do not need to be followed. **a** Axial contrast-enhanced CT showing a right renal too small to characterize mass (TSTC) (arrow); the renal mass is 8 mm in size, and the CT slice thickness is 5 mm, making the renal mass size less than twice the reconstructed CT thickness (8 mm/10 mm). **b** Axial contrast-enhanced CT showing a hypodense left kidney lesion measuring 3 HU (less than 20 HU), which is likely a simple cyst (arrow). **c** Axial non-enhanced CT showing a hyperdense left kidney cyst with an attenuation value of 85 HU greater than 70 HU, consistent with a hemorrhagic cyst (arrow). **d** Contrast-enhanced US of a Bosniak I cystic lesion showing no wall thickening or septa (arrow). **e** Unenhanced (right) and contrast-enhanced (left) US of a renal Bosniak II cystic lesion with thin septa that did not enhance in the post-contrast study (arrows)

Most renal masses incidentally found by cross-sectional imaging are cysts which, according to the Bosniak renal cyst classification system, do not require follow-up [13]. In 2017, the Incidental Findings Committee (IFC) of the American College of Radiology (ACR) updated its recommendations on the management of incidental renal masses by computed tomography (CT) [14]. This excellent guideline describes the different scenarios of the management of incidental renal masses. It is of note that in these guidelines, the following renal masses presumed to be non-complicated cysts and considered benign by imaging techniques do not require follow-up or treatment (Fig. 3).

- Homogeneous well-defined, too small to be characterized renal masses (TSTC) supposed to be cysts. Too small to characterize renal masses occur when the lesion size is less than twice the reconstructed slice thickness (Fig. 3a).
- Homogeneous well-defined renal masses with attenuation − 10 to 20 Hounsfield units (HU) by CT are likely benign cysts (Fig. 3b).
- Homogeneous well-defined renal masses with attenuation ≥ 70 HU on non-contrast CT consistent with hemorrhagic cysts (Fig. 3c).
- Completely characterized Bosniak I cysts diagnosed by ultrasound (US), CT, or magnetic resonance imaging (MRI) (Fig. 3d).
- Completely characterized Bosniak II cysts diagnosed by US, CT, or MRI (Fig. 3e).

**Fig. 4:**
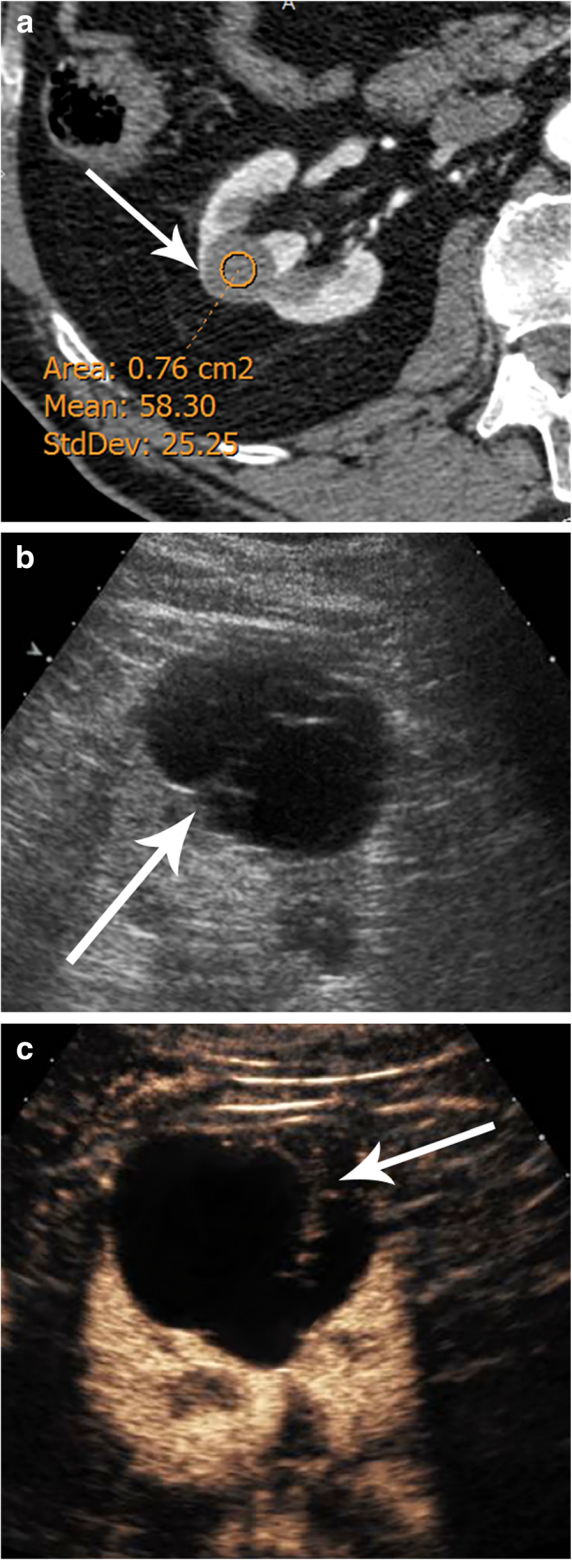
Indeterminate renal mass by CT in the right kidney. **a** Axial CT in the arterial phase depicts a renal tumor with 58 HU consistent with the indeterminate lesion (arrow). **b** Unenhanced and (**c**) contrast-enhanced ultrasound (US) of the same renal tumor showing the presence of a cyst with thin septa with no contrast uptake corresponding to a Bosniak II lesion (arrow): follow-up was not required

**Fig. 5:**
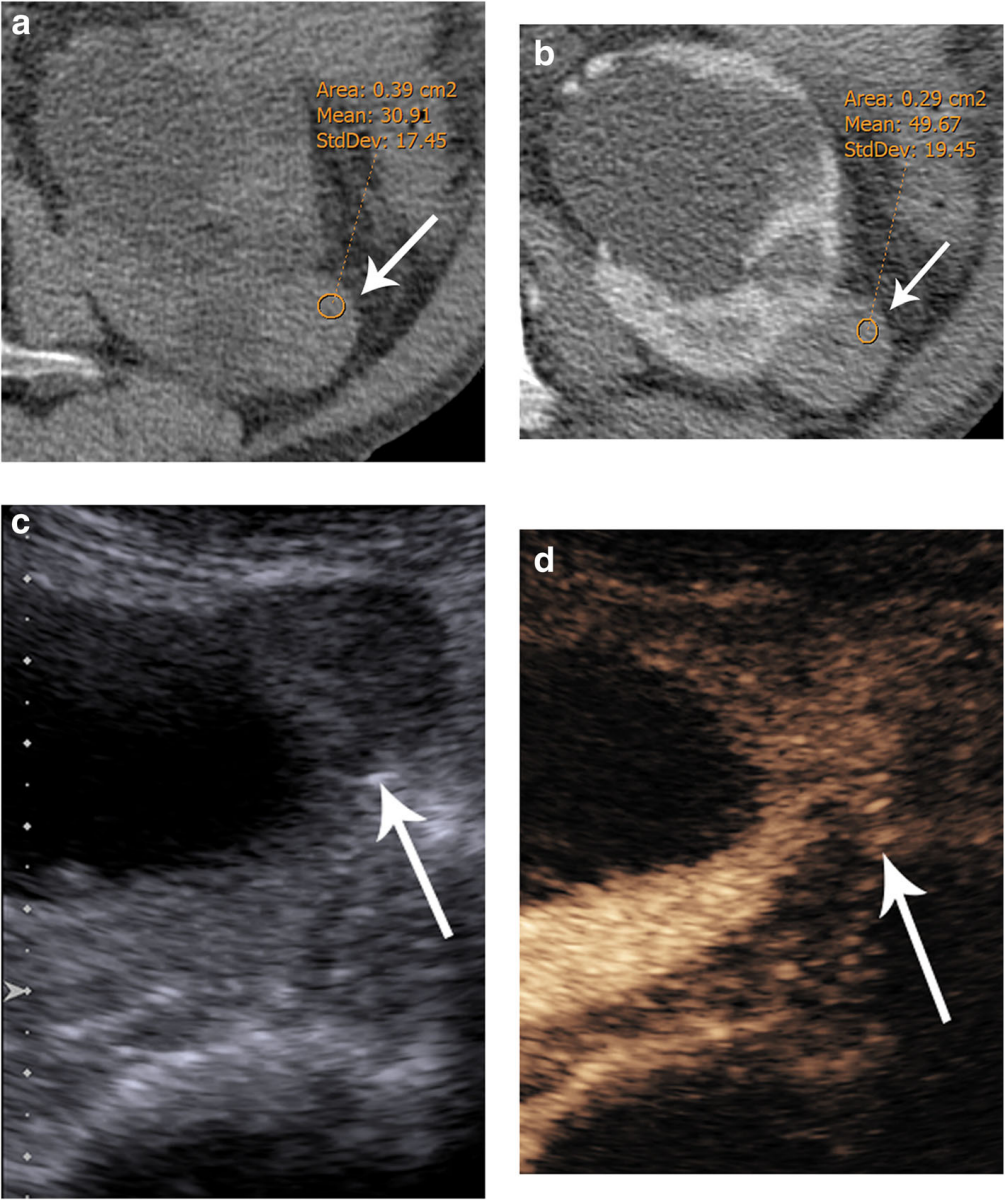
Indeterminate renal mass by CT in an 82-year-old patient. **a** Axial unenhanced and (**b**) enhanced CT showing a renal mass with attenuation greater than 20 HU in unenhanced CT that enhances less than 20 HU after contrast administration, considered indeterminate by CT (arrows). **c** Unenhanced and (**d**) contrast-enhanced ultrasound (US) of the same lesion clearly depicting enhancement of this lesion consistent with a solid renal tumor (arrows), thereby allowing an indeterminate CT renal mass to be reliably classified as a solid mass

The use of contrast-enhanced ultrasound (CEUS) can help to differentiate benign renal lesions and thereby increase the number of lesions that do not need to be followed. In our clinical practice, usually, we face only an enhanced CT with a renal mass measuring more than 20 HU, which is considered indeterminate. CEUS plays an important role in converting these indeterminate CT lesions into benign Bosniak I/II cysts that do not require further examination [15–17] (Fig. 4). On the other hand, some low-grade RCCs, especially the papillary subtype, show very slight enhancement which may not be clearly detected by CT or MR [18]. CEUS has shown to be an excellent tool in demonstrating blood flow within these hypovascular tumors (Fig. 5).

**Fig. 6:**
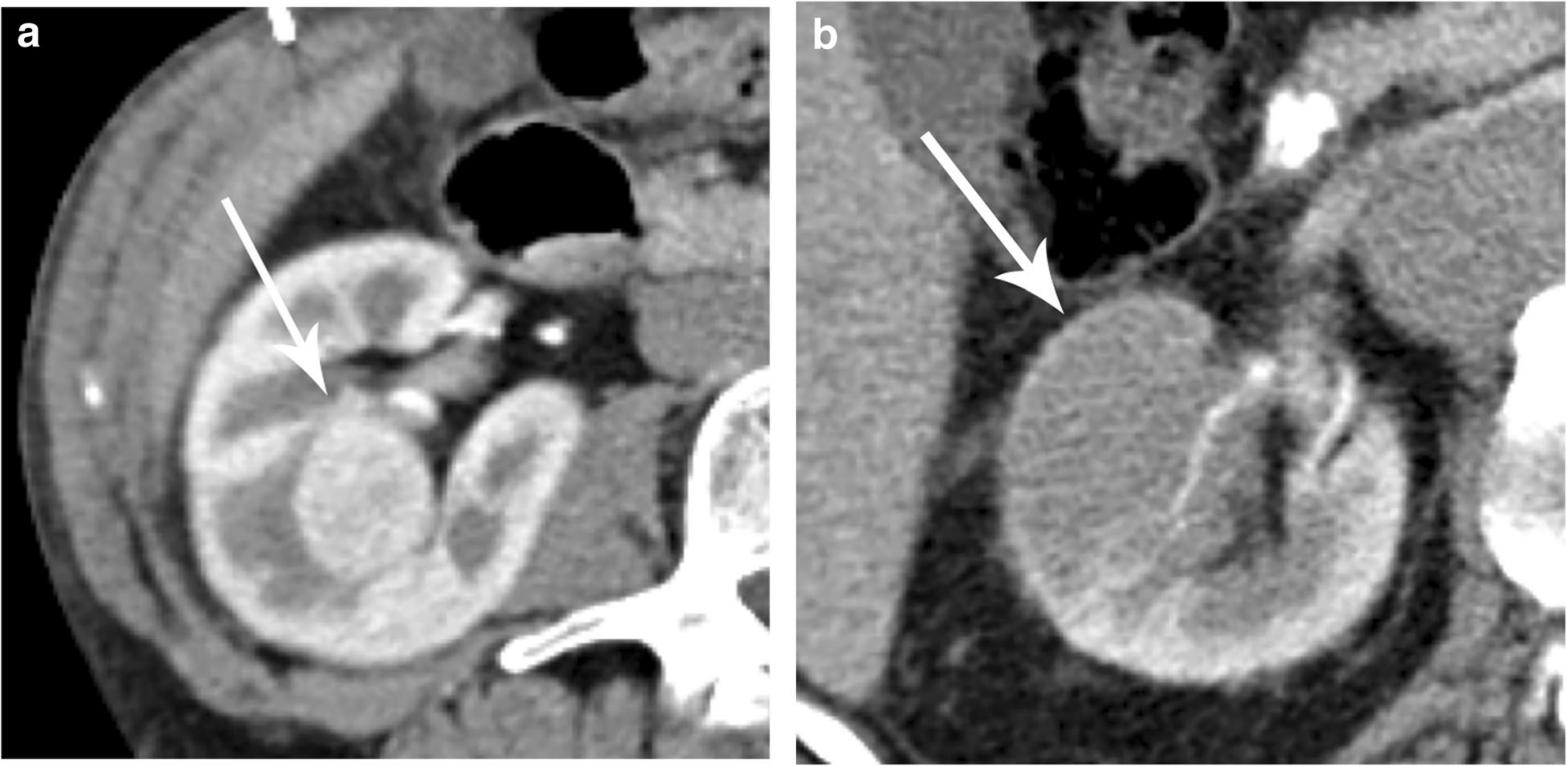
Solid renal masses in two elderly patients with regular and irregular borders. **a** Axial contrast-enhanced CT in the arterial phase shows a hyperdense right renal mass with smooth margins and regular shape (arrow) which, if indicated, can be managed with active surveillance. **b** Axial contrast-enhanced CT scan in the nephrographic phase depicting a hypodense renal mass with irregular borders (arrow). Active surveillance is not indicated in this case

AS is indicated only in SRMs which are regular in shape since masses with irregular borders are highly suspicious of being an aggressive tumor (Fig. 6). Renal masses with fat attenuation areas by CT or MR (macroscopic renal fat) without associated calcification are consistent with angiomyolipomas and must be managed separately and not by AS [14].

Localized renal masses mean that the tumor is contained by the capsule, independently of the size. If the tumor invades the capsule, the sinus, the renal vein, and the perirenal fat or has positive lymphatic or hematogenous spread, it is not considered a localized tumor, and AS is not an option [19].

## When to perform a biopsy in AS

In order to differentiate between benign and malignant tumors in the setting of incidental renal masses, radiological characteristics can be crucial. In this setting, many studies are focused on radiologic findings trying to correlate them with histological classifications. Currently, radiologists can properly characterize cysts or fat tumors consistent with angiomyolipoma as mentioned previously, but other renal mass histology cannot be predicted with 100% accuracy [20–22].

A quarter of SRMs are benign renal cortical tumors, such as oncocytoma, metanephric adenoma, and angiomyolipoma. Another 25% of these SRMs are indolent in nature with limited metastatic potential, including chromophobe, and type I papillary renal cancer [23]. Without histological assessment, the only parameter that helps to determine the aggressivity of these SRMs is their growth rate [24].

The prediction of biological tumor behavior plays an important role in choosing the most appropriate treatment in each case, AS being more adequate for benign and indolent renal tumors. All the current guidelines agree that renal tumor biopsy (RTB) should be performed, if clinically and technically possible, in cases in which the results might alter treatment [12]. According to the European Urology Association guidelines, RTB should only be performed before ablative or systemic therapy, while it is considered less necessary in AS (https://uroweb.org/guideline/renal-cell-carcinoma) [25]. 

As for the histological predictive factors of the biological behavior, the European urological guidelines include tumor grade, RCC subtype, sarcomatoid features, microvascular invasion, tumor necrosis, and invasion of the collecting system. Currently, there are some molecular factors described such as carbonic anhydrase IX (CaIX), VEGF, hypoxia-inducible factor (HIF), Ki67, p53, and p21, but none of these are routinely used [26].

Currently, RTB, and specifically core biopsy, is the only reliable way to confirm the diagnosis of renal masses without surgery. A recent systematic review summarizing RTB results reported a median overall diagnostic rate of 92% with a sensitivity and specificity of 99.7% and 93.2%, respectively [23].

Although RTB may be helpful in achieving the diagnosis of renal tumors, in around 10 to 20% of the cases, this procedure is non-diagnostic. An oncocytic feature designation can be controversial because of the possibility of benign or malignant variant lesions. Moreover, tumor heterogenicity in terms of morphology, grade, and molecular characteristics is well recognized in renal tumors [27]. Nonetheless, two recent articles reported that the genetic heterogeneity of SRMs is not as great as previously thought [28, 29].

On the other hand, significant complications may occur in 5% of patients undergoing RTB, usually related to the development of perinephric hematomas. Seeding in the needle tract is in fact an extremely rare complication, with no cases described using the coaxial technique [30, 31].

SRMs with irregular borders should be treated and should not be included in AS because of the high probability of aggressive malignancy. After having ruled out inflammatory and infectious masses, RTB should be performed to determine the possible presence of metastatic disease, urothelial carcinomas, and aggressive RCCs and lymphomas.

RTB is not performed in young or healthy patients, who are unwilling to accept the uncertainties associated with AS, or in older or frail patients who receive conservative treatment, independently of RTB findings [4].

## Active surveillance protocol

When AS is determined to be the primary management option of a SRM, a strict follow-up imaging protocol must be carried out.

There is a consensus between guidelines as to the optimal imaging follow-up schedule. However, all guidelines agree that close follow-up is necessary during the first 2 to 3 years of AS, since most M1 appear during this period. In addition, the tumor growth rate (GR) is more undetermined in the first years [8].

**Fig. 7:**
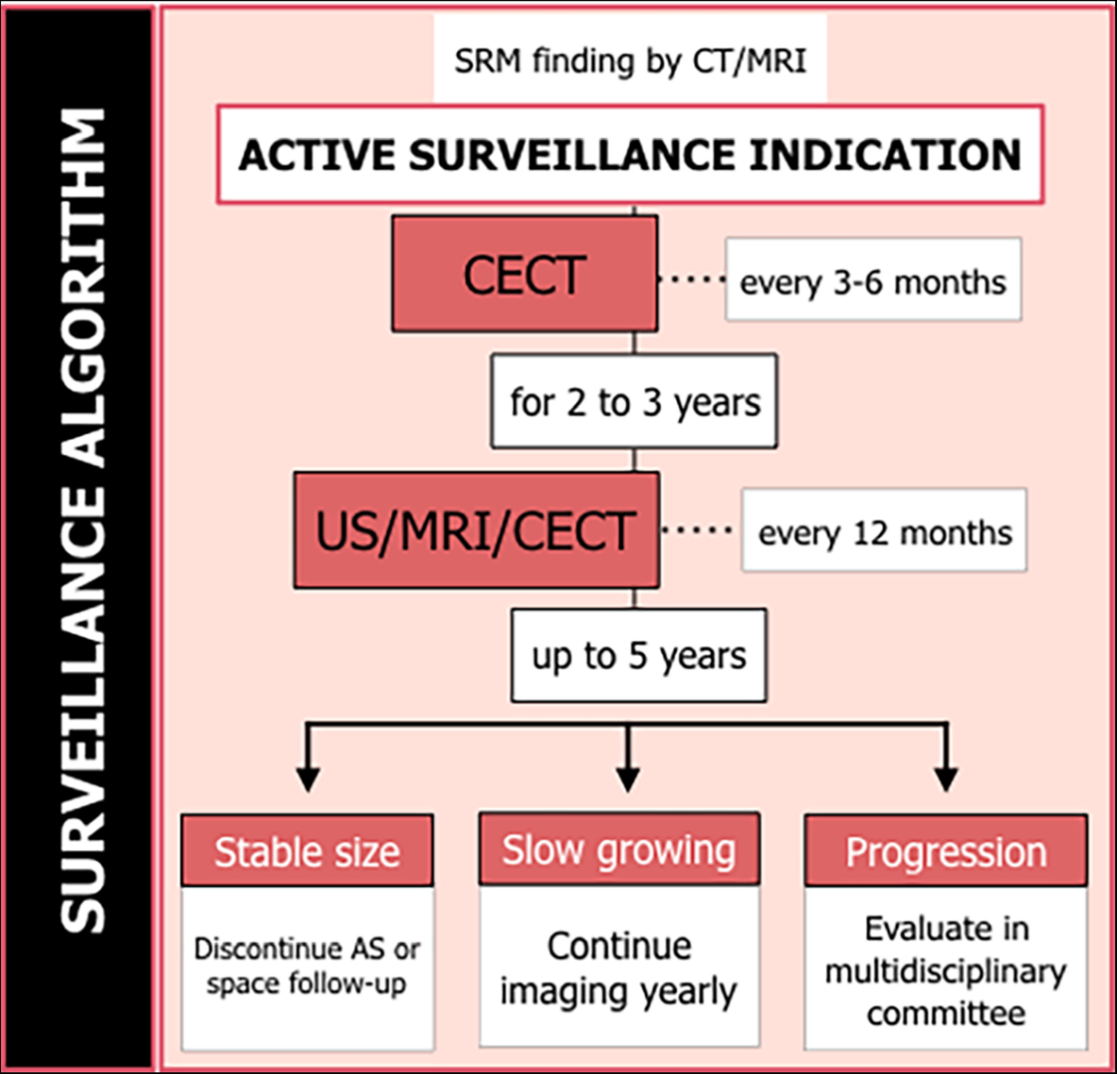
Suggested active surveillance management algorithm. Contrast-enhanced computerized tomography (CECT), ultrasound (US), and magnetic resonance (MR)

The follow-up protocol most commonly used includes enhanced abdominopelvic CT scanning at 3–6-month intervals during the 2 or 3 first years and yearly thereafter. CT can be substituted by MR or US, especially after the 2 first years, to avoid radiation or in cases in which iodine contrast agents are contraindicated [12]. A complementary annual chest X-ray is also recommended in many guidelines [10] The surveillance algorithm of the National Comprehensive Cancer Network (NCCN) recommends the use of CT or MR of the head or spine in patients with SRM presenting neurologic symptoms or a bone scan in cases with elevated alkaline phosphatase values, bone pain, or abnormal radiologic findings [32]. A proposed follow-up AS algorithm is shown in Fig. 7.

**Fig. 8:**
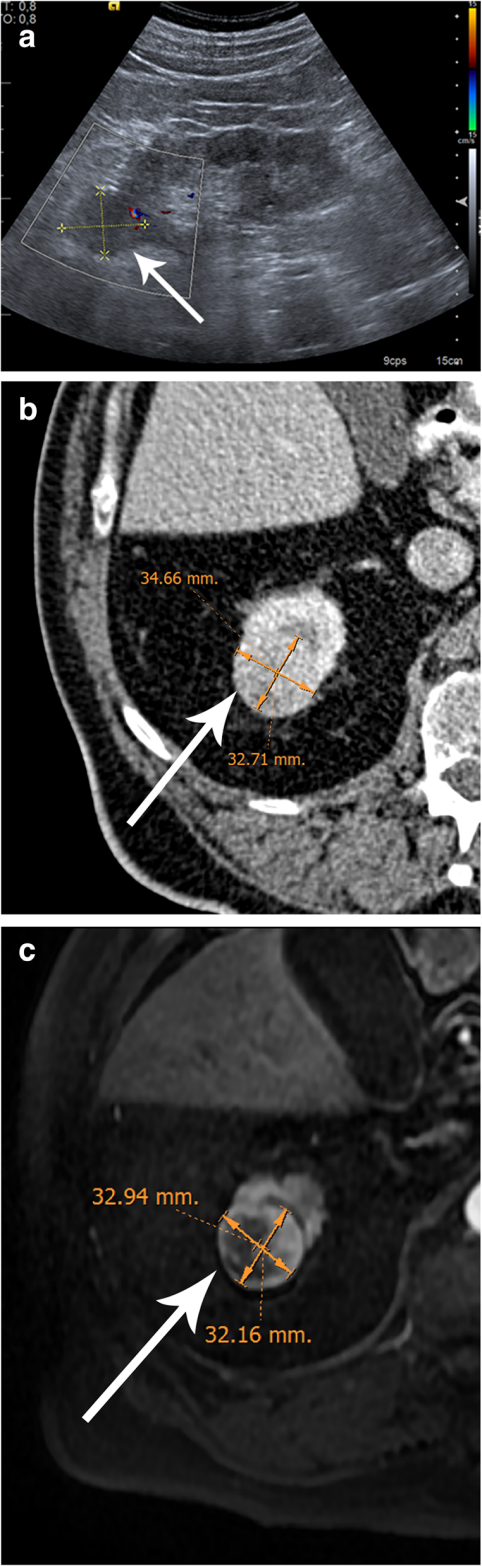
Comparison of maximum tumor diameter (MTD) in the axial plane of a small renal mass (SRM) with different imaging modalities (arrows). **a** Sagittal ultrasound (US) view. **b** Axial contrast-enhanced CT scan and (**c**) axial contrast-enhanced T1-weighted MR of the same renal lesion demonstrating there are no significant differences between US, CT, and MR with the use of the same plane for measuring MTD (3.33 cm, 3.46 cm, and 3.29 cm, respectively). Note that the differences between different radiological tests are less than 0.2 cm

**Fig. 9:**
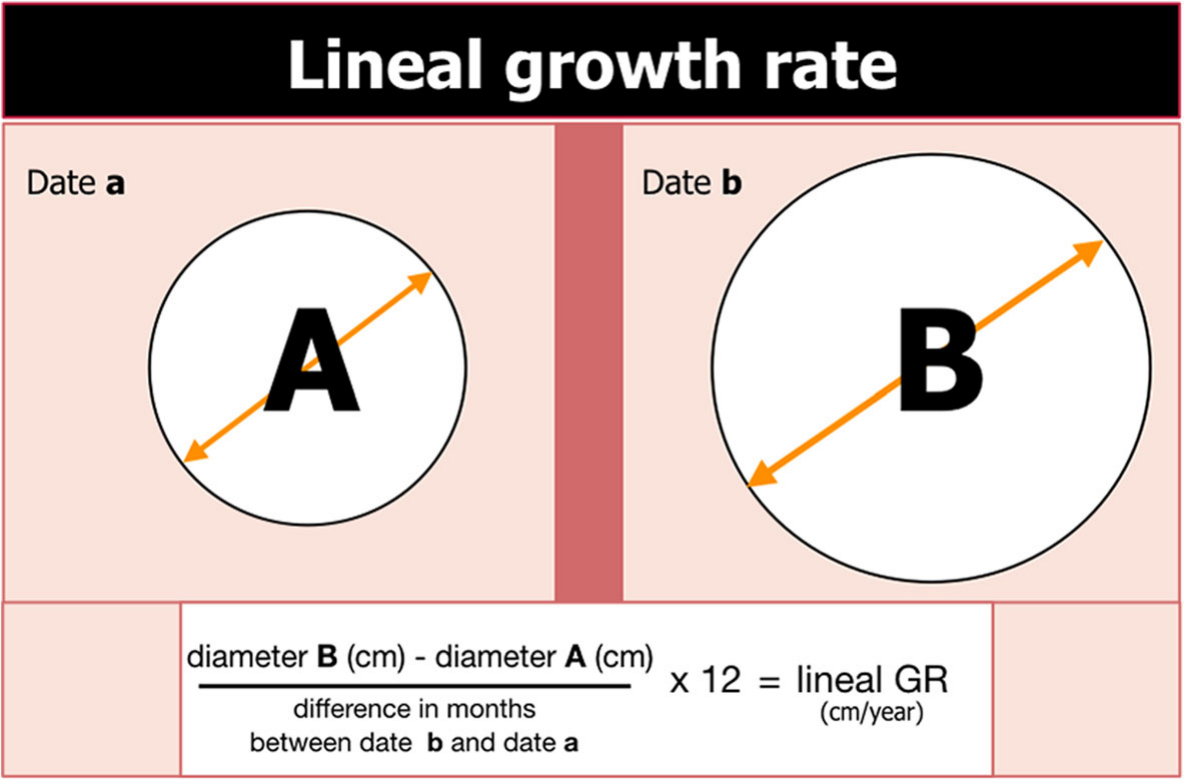
Formula of linear growth rate (GR) per year. To define the linear GR per year, divide the difference between the maximum tumor diameter (MTD) into two time points by the difference of the number of months between the two CT times and then multiply by 12

In most of the studies comparing preoperative US, CT, and MR results, the differences between the final kidney tumor size are minimal [33–36] (Fig. 8). The maximum tumor diameter (MTD) is the easiest way to measure SRMs, although the cross-sectional area and volume can also be measured. To define the linear GR, you divide the difference between MTD into two time points and the number of months passed and then multiply by 12, to obtain the GR/year [37] (Fig. 9).

**Fig. 10:**
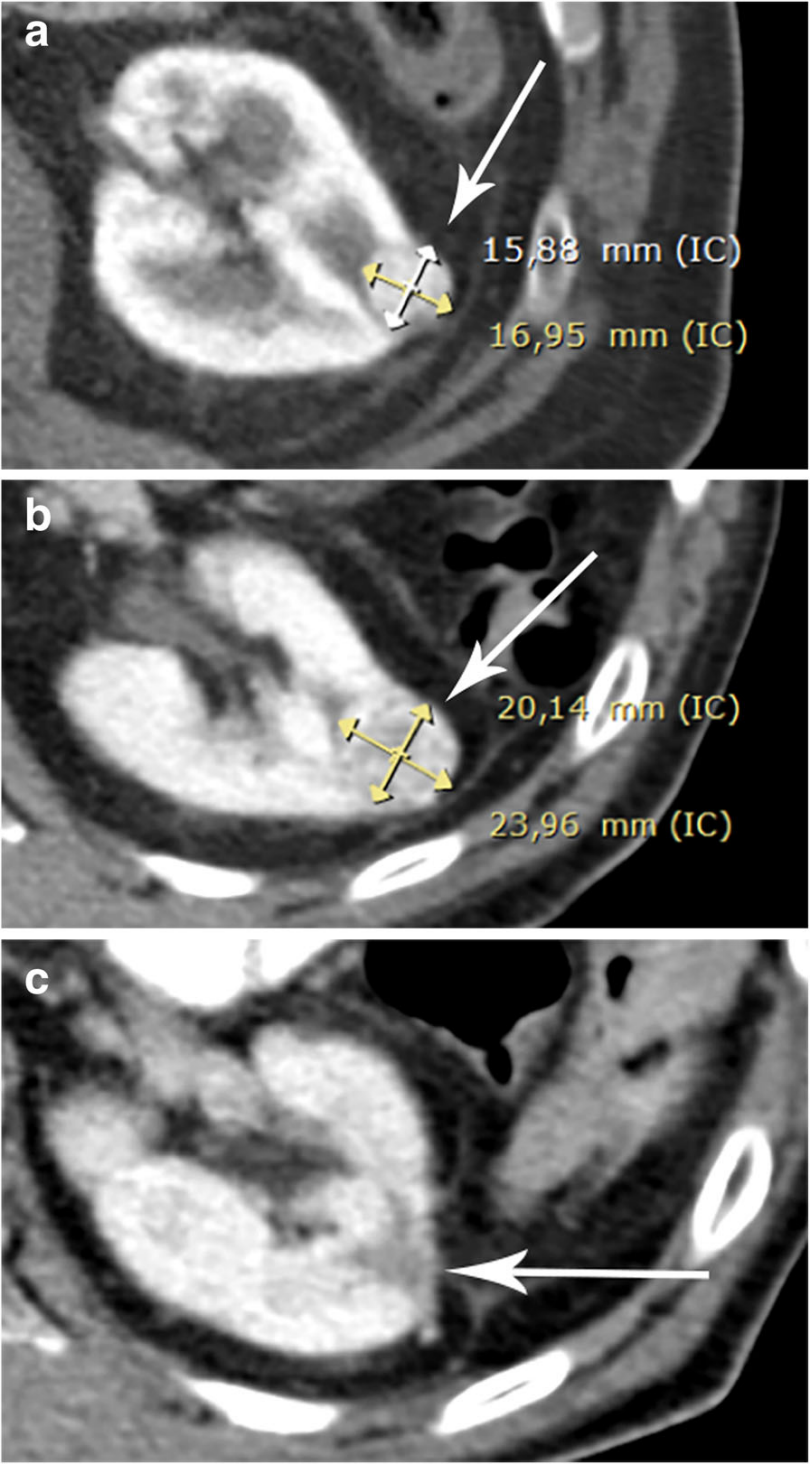
Linear GR calculation of a SRM in AS in a 73-year-old patient. **a** Contrast-enhanced CT of a renal mass (arrow) with a MTD of 1.7 cm (16.95 mm) at the beginning of AS and (**b**) 3 months later (arrow), showing a MTD of 2.4 cm (23.96 mm). Using the linear GR formula, the difference of MTD (2.4 cm − 1.7 cm = 0.7 cm) is divided by the time between the two CT scans (3 months), and the result (0.3 cm) is multiplied by 12, resulting in 2.6 cm. **c** Taking into account that the mass presented a GR of more than 0.5 cm/year, this mass should be considered as progressive, and AS should be discontinued. Following evaluation by a multidisciplinary committee, the patient underwent partial nephrectomy (arrow)

**Fig. 11:**
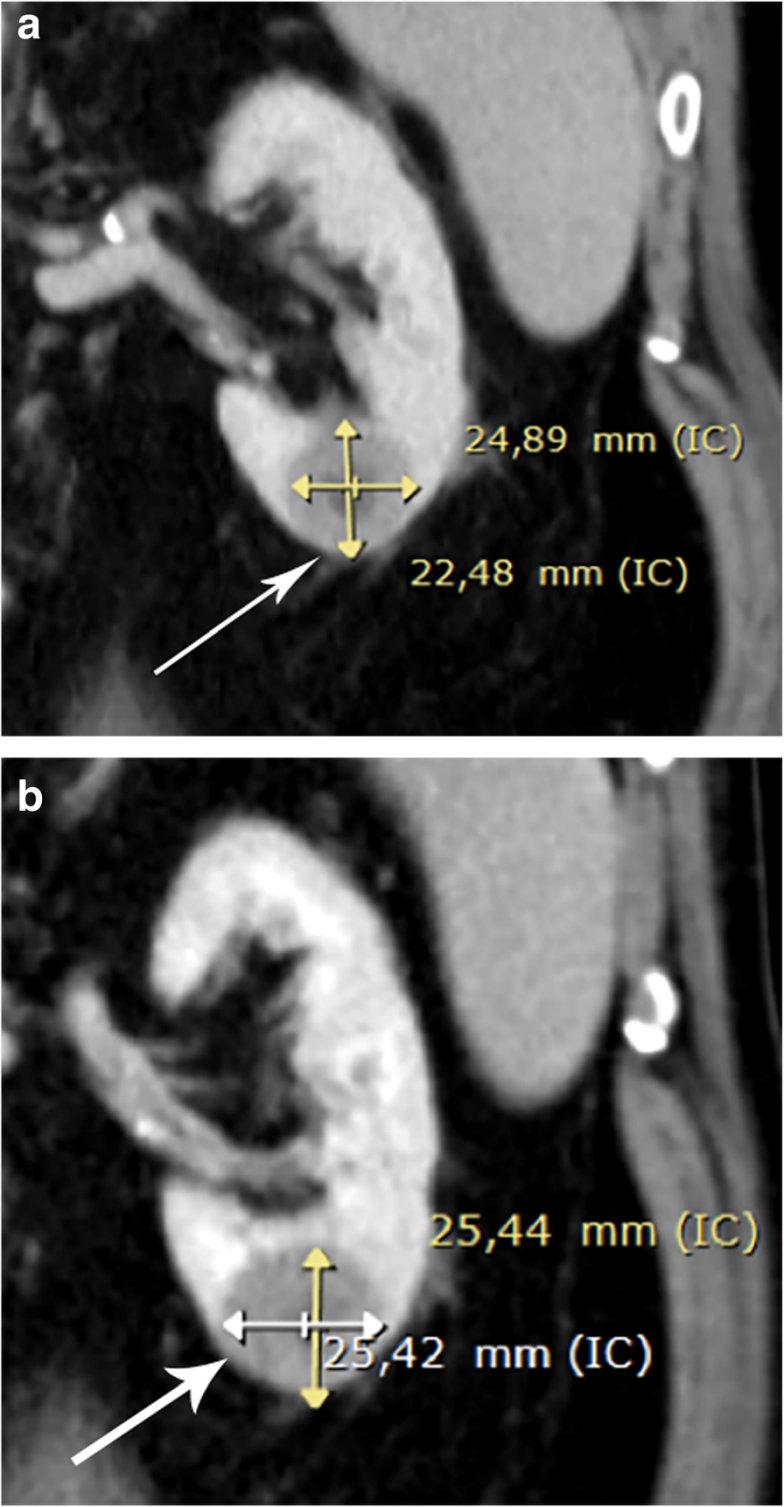
Calculation of the linear growth rate (GR) of a lower third left kidney small renal mass (SRM) undergoing active surveillance (AS) in a 65-year-old patient (arrows). **a** Coronal contrast-enhanced CT of the renal mass showing an axial MTD of 2.5 cm (24.89 mm) at the beginning of the surveillance and (**b**) 3 which had only increased to 2.5 cm (25.44) 3 years later. With the use of the linear GR formula in centimeters, 2.5 cm − 2.5 cm = 0 cm divided by 36 months and multiplied by 12, the annual GR was shown to be 0 cm/year, indicating that the renal mass was stable. After 5 years of follow-up, the interval between imaging tests can be lengthened or even discontinued according to the decision of the multidisciplinary committee

The linear GR is a universally available and easy AS method of SRMs. Several studies have shown the linear GR to have a good correlation with tumor aggressiveness to guide patient follow-up and be a potential determinant for intervention [38–42]. However, not all studies have shown an association between the tumor GR and the characteristics of high-risk tumors. The tumor GR alone is not a sufficient predictor of malignancy because benign masses can grow at a similar rate to that of malignant lesions [37, 43]. Despite the ongoing debate of predictors of GR and tumor aggressiveness, the majority of the literature supports the concept that larger tumor size and a fast GR are associated with more aggressive disease. Therefore, it is currently recommended that tumor size greater than 4 cm and tumor growth of greater than 0.5 cm/year be used as a marker of disease progression and for establishing definitive treatment [44] (Fig. 10). Nonetheless, it must also be acknowledged that a proportion of masses display no growth or even regress in size. The proportion of SRMs with negative or zero growth ranges from 10 to 25% in different series [42, 45, 46] (Fig. 11), and in these cases, patient follow-up with imaging techniques can be less frequent (every 2–3 years) or even discontinued after 5 years of documented stability.

The slow relative GR of SRMs and the risk of intra- and interobserver variability require accurate radiographic measurement to establish subtle changes in mass dimension [47]. The use of mixed modalities of imaging such as US, CT, and MR is used in the follow-up of SRMs, measurement accuracy diminishes, although the differences between US, MR, and CT results are only of 0.1–0.2 mm [48].

When a SRM is followed by AS, the same imaging modality should be used throughout the AS in order to avoid the variability in measurement between different techniques, at least during the first 2 to 3 years. Indeed, enhanced CT is recommended in these cases because of its reproducibility and higher spatial resolution. It is also important to measure the lesion on the same plane, in the same direction, and in the same slice, recommending the nephrographic phase to reduce variability. It is recommended to record the image with a size caliper and compare it from one test time to another, or report the slice and the sequence used for measurement using response evaluation criteria in solid tumor (RECIST) measurements.

**Fig. 12:**
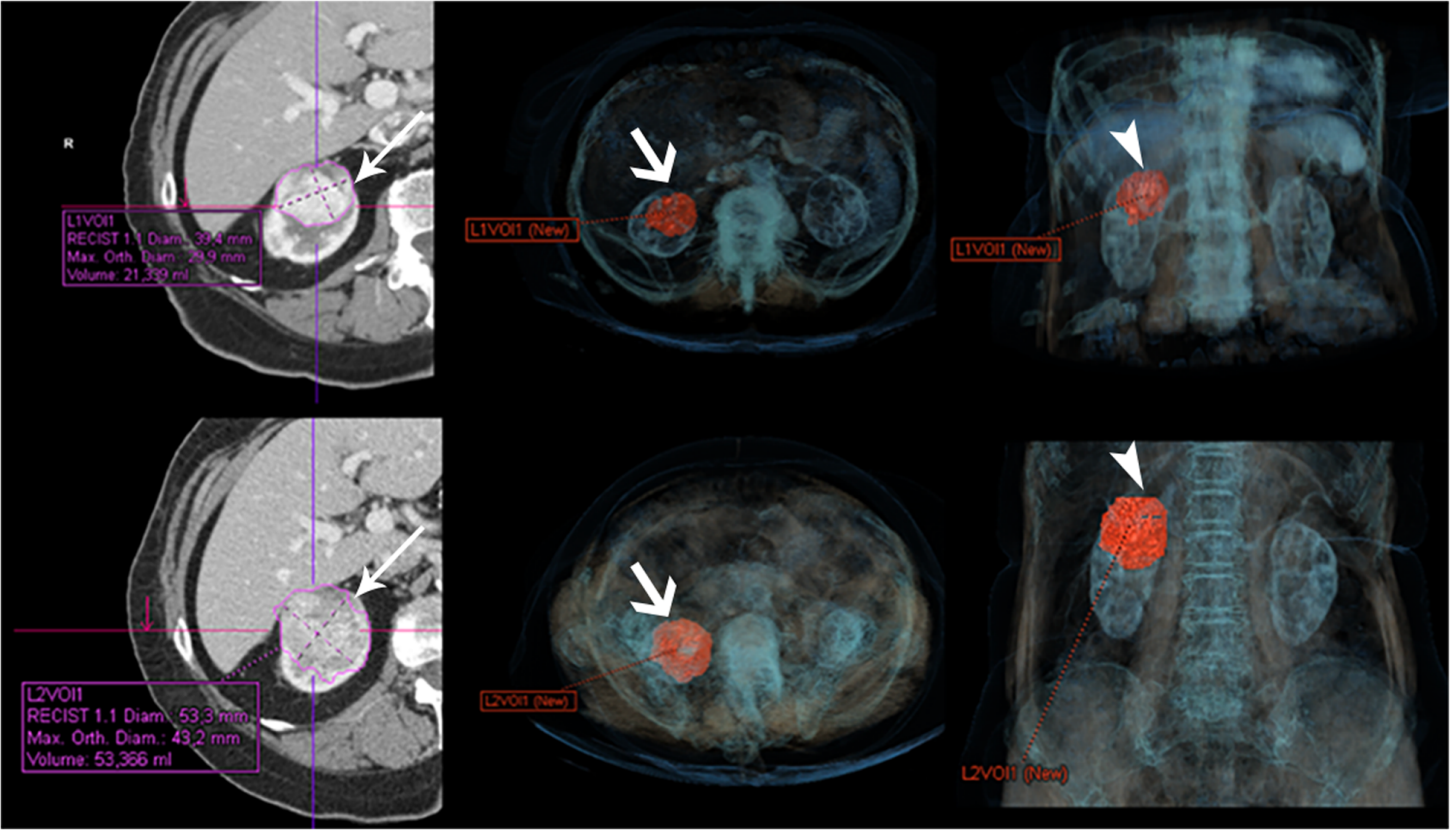
Comparison between linear and volumetric measurements of small renal mass (SRM) growth over a 6-month period. Upper- and bottom-left: axial contrast-enhanced CT showing a renal mass with MTD 3.9 cm at the beginning of AS and 5.3 cm in the next study 6 months later with a linear GR of 1.4 mm between the two studies corresponding to a linear GR of 3.8 cm/year (arrows). Volumetric representation of the renal mass in an axial plane (thick arrows) (upper- and bottom-middle) and coronal plane (arrowheads) (upper- and bottom-right). The software showed an initial volume of 21 cc and a final volume of 53 cc. The increase in volume was 32 cc, indicating that the mass had doubled in volume over a 6-month period representing a rapid growth, although there are no established volume values to define progression

Volumetric measurement is more accurate than axial diameters and can detect smaller growth. However, the reconstruction of these images requires more time as well as specific software which may not be available in all centers (Fig. 12).

**Fig. 13:**
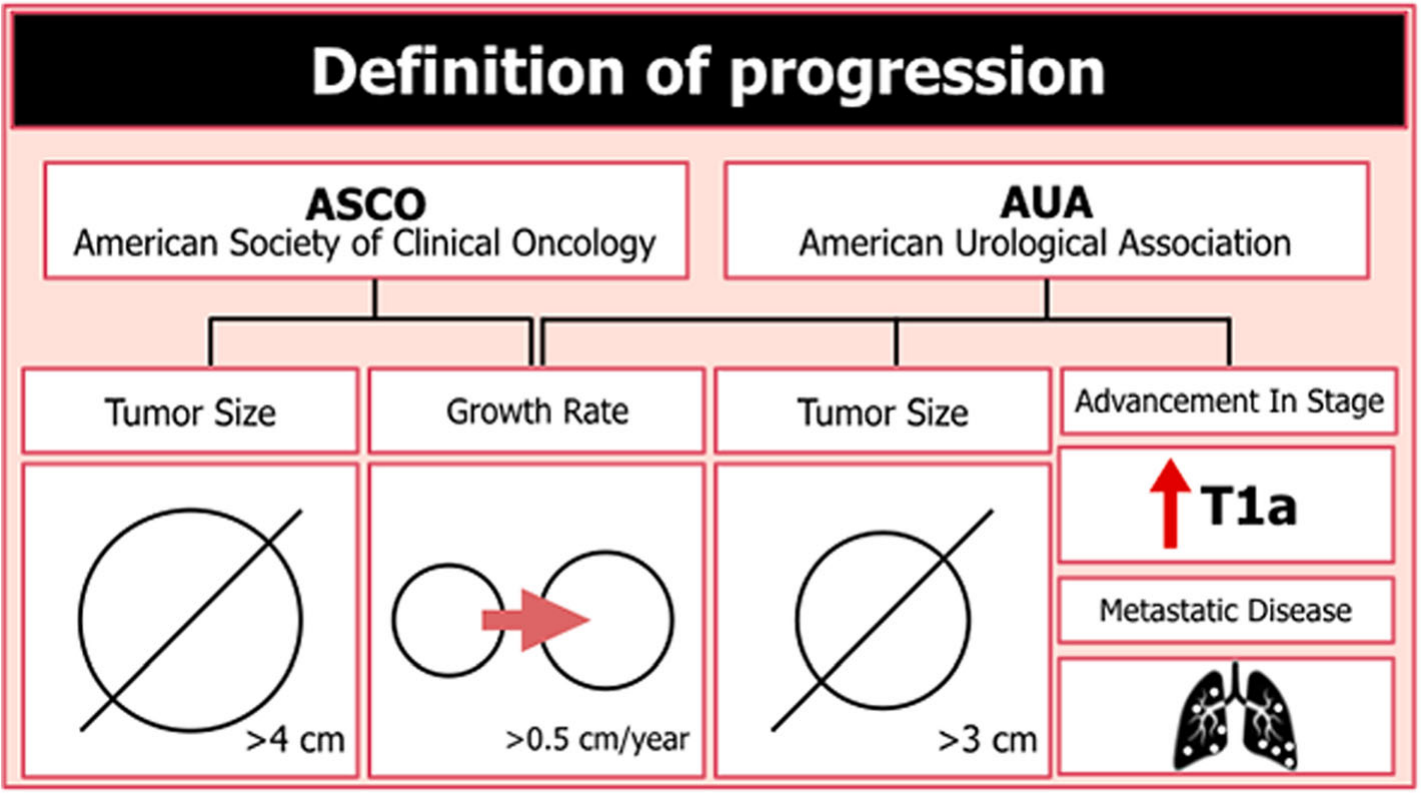
Definition of active surveillance progression according to the American Society of Clinical Oncology (ASCO) and American Urological Association (AUA) guidelines

AS should be discontinued on the progression of SRM, when the patient wishes active treatment or because of changes in the clinical situation. Progression is defined as linear GR greater than 0.5 cm per year, a tumor diameter greater than 4 cm, or the development of metastasis (Fig. 13) [2, 4]. Patients undergoing AS can, at any time, decide to initiate active treatment or may also choose to remain on AS despite having progressed [45]. In stable SRMs, follow-up can be discontinued or lengthened after 5 years, although there is no consensus in urological or oncological guidelines regarding which cases and at what time follow-up can be safely discontinued.

## Complex renal cysts and active surveillance

**Fig. 14:**
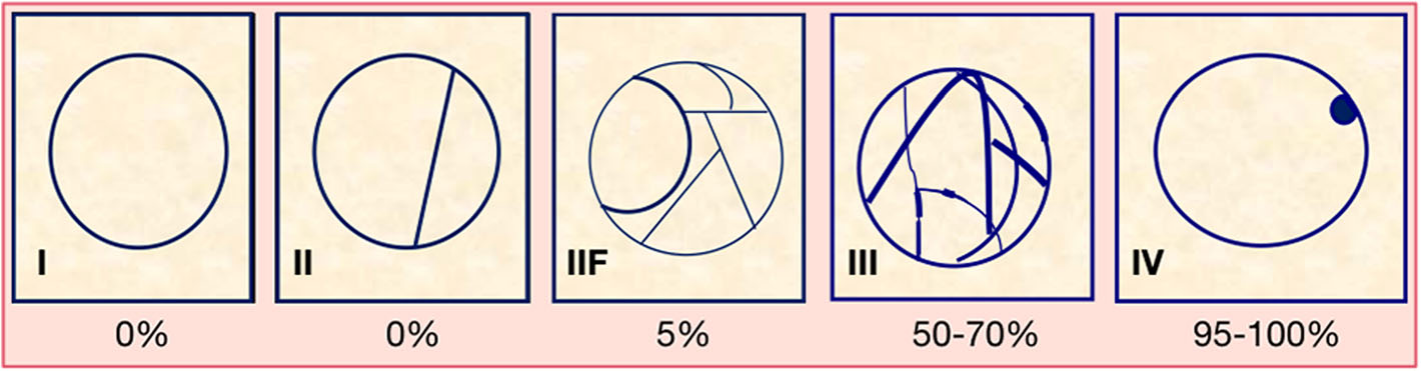
Schematization of Bosniak’s classification for cystic renal masses and associated malignancy rate. Bosniak I: hairline-thin wall without septa, calcifications, or solid components; malignancy rate of 0%. Bosniak II: few hairline-thin septa, fine calcifications in a short segment of the wall, or slightly thickened calcification; malignancy rate of 0%. Bosniak IIF: multiple hairline-thin septa, smooth minimal thickening of the wall or septa, and thick or nodular calcifications; malignancy rate of 5%. Bosniak III: thickened irregular wall or septa with enhancement after the administration of contrast agent; malignancy rate of 50–70%. Bosniak IV: soft tissue enhancing mass independent of the wall or septa; malignancy rate of 95–100%. Remember that Bosniak I–II cysts do not need follow-up, Bosniak IIF cysts need follow-up, and surgery is indicated for Bosniak III–IV cysts

With the increasing incidental identification of solid SRMs, the identification of complex cystic renal masses has increased by 8 to 15% in renal cysts [49]. The complexity of renal cysts is classified using the Bosniak renal cyst classification system, which was first applied in CT and MR and more recently in CEUS [13, 16]. The Bosniak classification or determination of renal cyst complexity was initially used to aid in differentiating non-surgical (categories I, 2II, and IIF) from surgical lesions (categories III–IV) according to the category of malignancy probability (Fig. 14). According to the Bosniak classification system, a IIF cyst should be followed, while surgery is indicated for Bosniak III and IV cysts, despite the knowledge that 50% of Bosniak III cysts are benign.

Cystic RCCs (multilocular cystic RCC, cystic RCC, and RCC with cystic degeneration) are usually low-stage and low-grade tumors with an excellent prognosis [13, 50, 51]. In a recent series of IIF and III cysts undergoing AS, none showed local progression or metastatic disease, making it unclear whether Bosniak III warrants the aggressive surgical treatment that is currently recommended. Therefore, it may be possible to offer AS to patients with progressive Bosniak IIF or Bosniak III as an alternative to surgery in certain clinical scenarios similar to that of patients with SRMs [43].

**Fig. 15:**
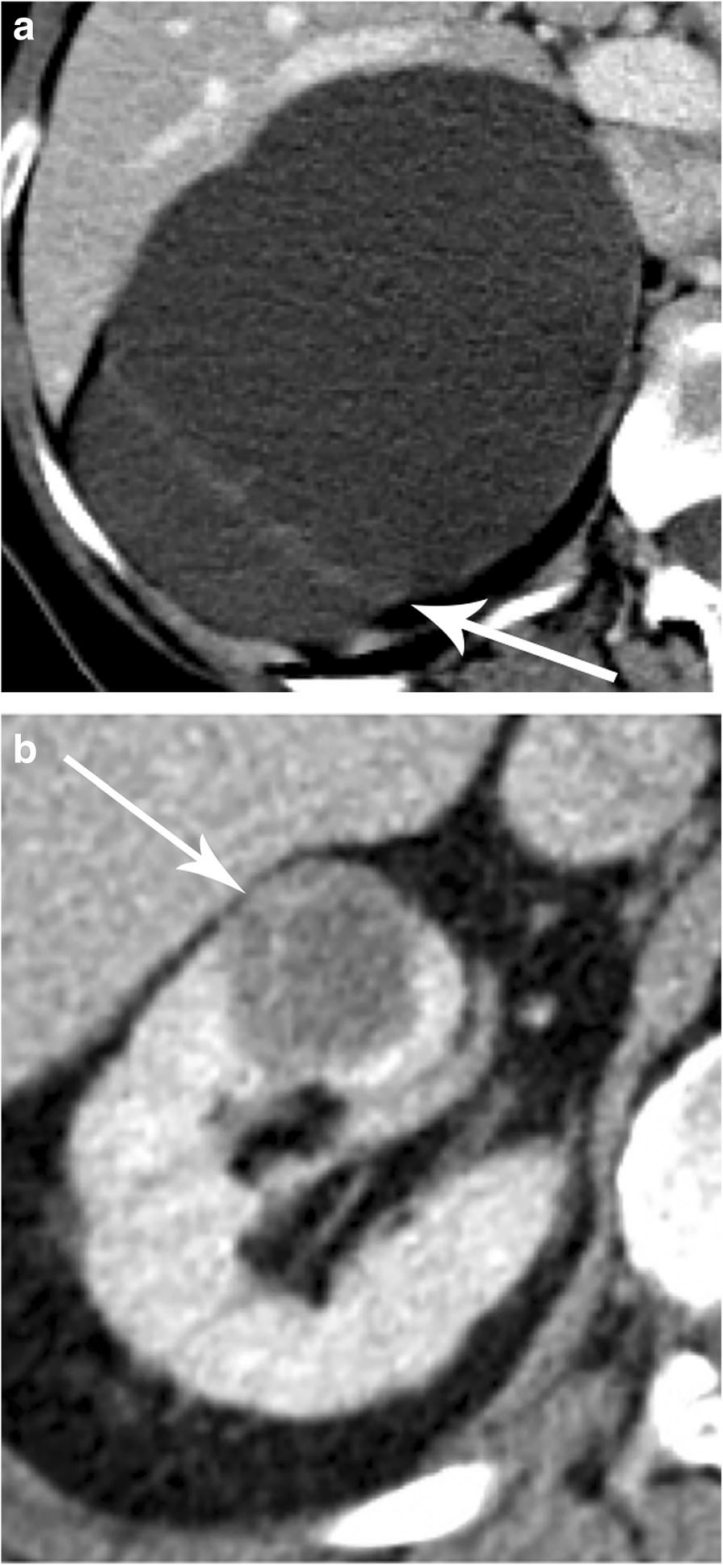
Bosniak IIF, differentiation between low and more complex lesions. **a** Axial contrast-enhanced CT depicting a large cyst with a solitary thick septum. The cyst should be followed because the septum is thick, but suspicion of malignancy is very low (arrow). **b** Axial contrast-enhanced CT demonstrating a cyst with multiple septa. This complex appearance does not completely rule out a possible cystic tumor (arrow)

The Bosniak IIF group included both less and more complex lesions (Fig. 15). In the former group, a follow-up of 1–2 years may be sufficient, while the latter group may require repeated imaging for at least 4 years [52]. More complex Bosniak IIF lesions that do not change within 2 years may be followed with another CT/MR 24 months later, and if no changes are observed, follow-up can thereafter be discontinued [43].

**Fig. 16:**
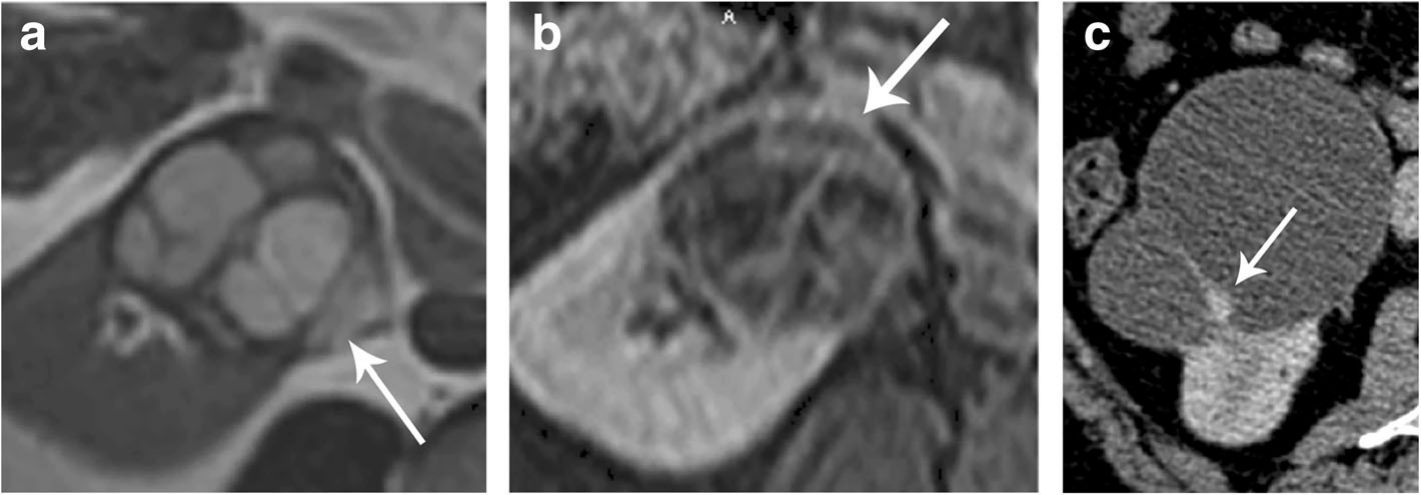
Bosniak IIIs (**a**, **b**) and IIIn (**c**) lesions. **a** Axial T2-weighted MR showing a multiseptated cystic renal lesion (arrow) and (**b**) axial T1 post-contrast MR of the same lesion showing septal enhancement (arrow) consistent with a Bosniak IIIs cyst. **c** Axial contrast-enhanced CT of a cystic renal mass with septae nodularity (arrow) considered to be a Bosniak IIIn cyst

Recently, Bosniak III cysts have been classified as Bosniak IIIs and IIIn. Bosniak IIIs cysts only have enhanced thickened septations, and Bosniak IIIn cysts have thickened walls or septal enhancement and nodularity, demonstrating that only Bosniak IIIn cysts are associated with progression (Fig. 16) [53]. In addition, in a recent study, a mural or septal nodule was associated with malignancy, but septation thickness, wall thickness, and cyst size were not found to be determinants of malignancy [54].

**Fig. 17:**
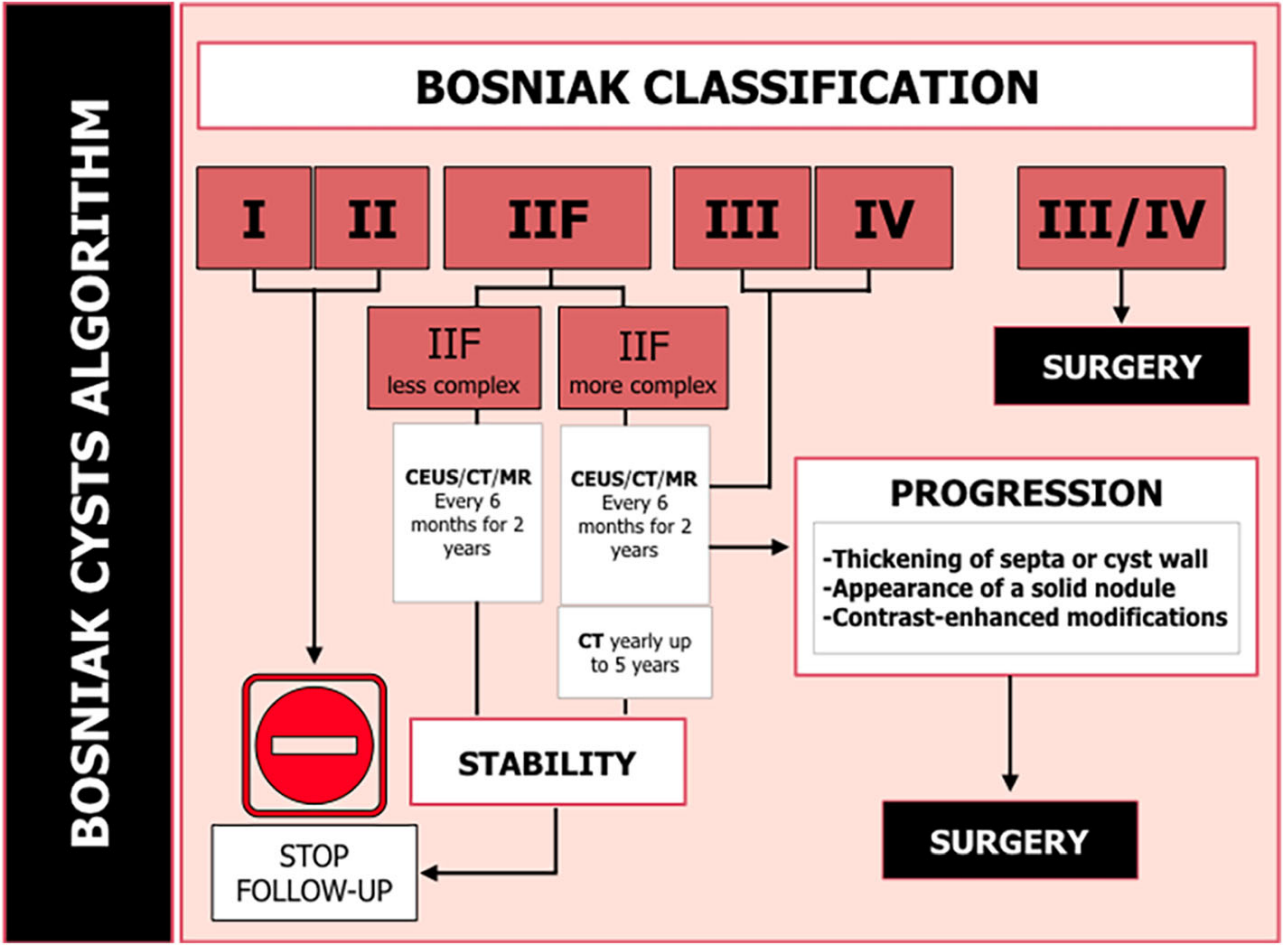
Suggested Bosniak cyst classification management algorithm

AS of complex renal cysts differs from that of solid masses. Figure 17 shows a possible follow-up algorithm for these lesions. The size of complex renal cysts has no correlation with the incidence of RCC or tumor aggressiveness, with a recent study reporting that malignant cysts are smaller than benign cysts [55]. During the follow-up of cysts, changes in the internal architecture such as septa and wall thickening or nodularity are taken into account to demonstrate progression [43]. The indolent behavior of cystic malignancies suggests that delayed intervention should be strongly considered when treating patients with cystic renal masses, even Bosniak IIIn and IV cysts in the same scenarios recommended in SRM guidelines.

However, Bosniak IV renal cysts can also be highly necrotic and aggressive solid renal neoplasms. There is limited experience with imaging surveillance of Bosniak IV renal lesions. In cases of AS of Bosniak IV tumors, if a solid nodular septum or nodular wall cannot consistently be found in order to correctly follow-up in terms of progression, we suggest managing it as a small renal mass (Fig. 7) and not as a cystic Bosniak IV in terms of progression. This means that in these cases, the correct follow-up procedure to assess progression is by checking the increase in diameter of the lesions [56].

**Fig. 18:**
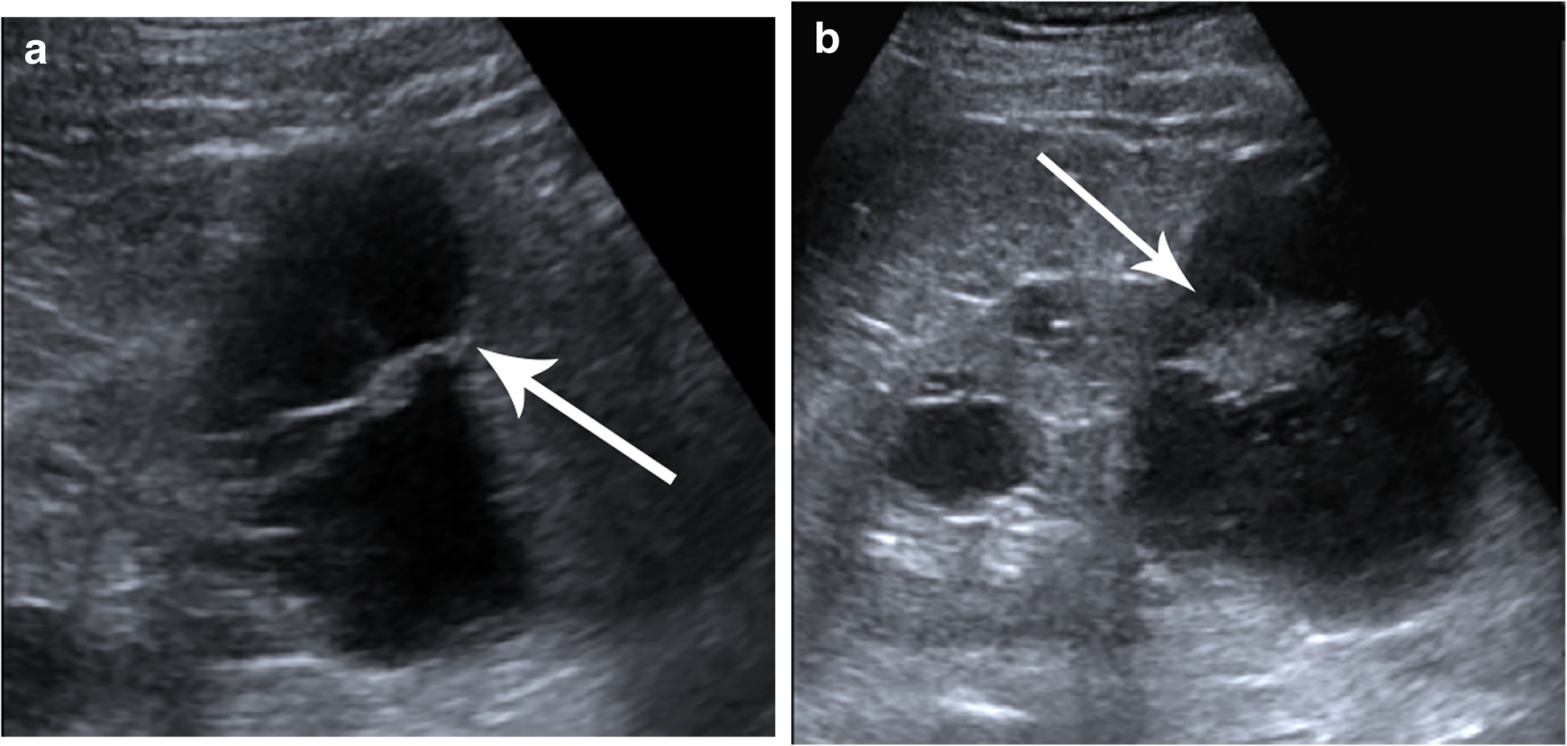
Cystic Bosniak IIF renal mass (arrows) presenting growth over time and showing malignant features with an occurrence rate of 5%. **a** Unenhanced US showing a cystic renal lesion with thickened septa. **b** Unenhanced US of the same lesion 1 year later showing increasing nodularity in the previously thickened septa, consistent with progression

**Fig. 19:**
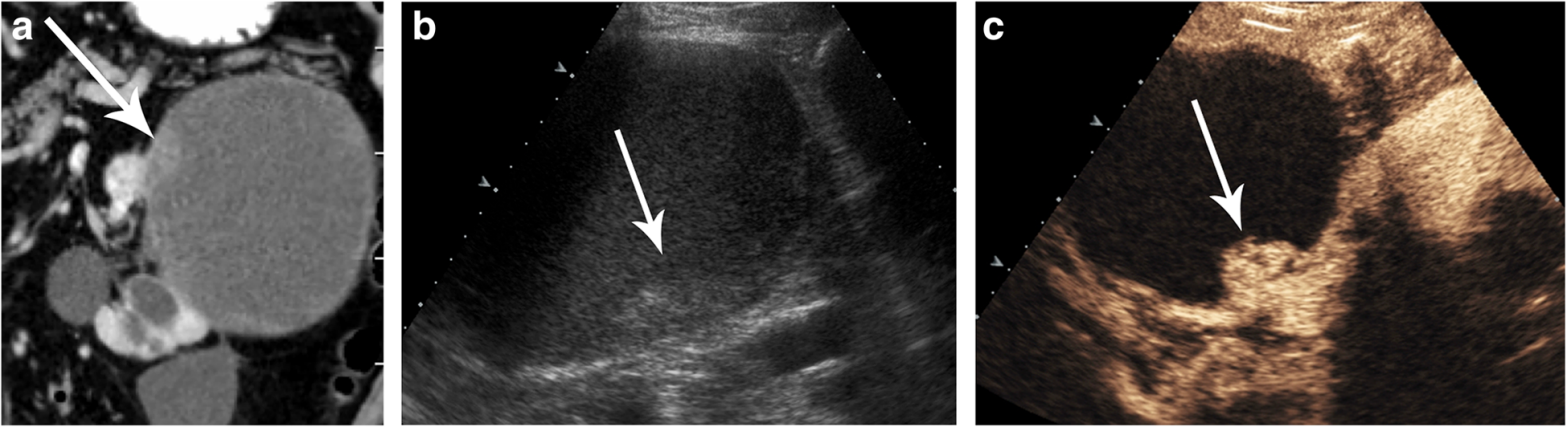
Contrast-enhanced US image showing a cystic renal lesion in the wall of the left kidney suspected of being a solid lesion. **a** Coronal contrast-enhanced CT showing a cystic renal mass with a hard-to-define image in its wall (arrow). **c** Unenhanced and (**d**) contrast-enhanced US clearly depicting enhancement on this solid nodule (arrows) in the cyst wall, leading to the lesion being defined and classified as a Bosniak II lesion

Recent studies have demonstrated the high accuracy of CEUS in the characterization of baseline complex cysts [57]. In the study of Quaia et al., CEUS performed better than CT in the diagnosis of malignancy in complex cystic renal masses, with a similar diagnostic reliability between CEUS and CT [28]. CEUS can also be used instead of CT in the follow-up of Bosniak IIF cysts to detect any morphologic changes considered as progressions such as septa thickening, the appearance of a solid nodule, or contrast-enhanced modifications indicative of disease progression with the additional benefits of a reduction in costs and radiation exposure. CEUS helps to demonstrate the enhancement of hypovascular nodularities of cysts, which are not well demonstrated by CT or US (Fig. 18). CEUS is also useful in hemorrhagic cysts diagnosed by CT or MR, classified as Bosniak IIF if bigger than 3 cm; if CEUS reclassifies these cases as Bosniak I–II, follow-up is not required (Fig. 19).

**Fig. 20:**
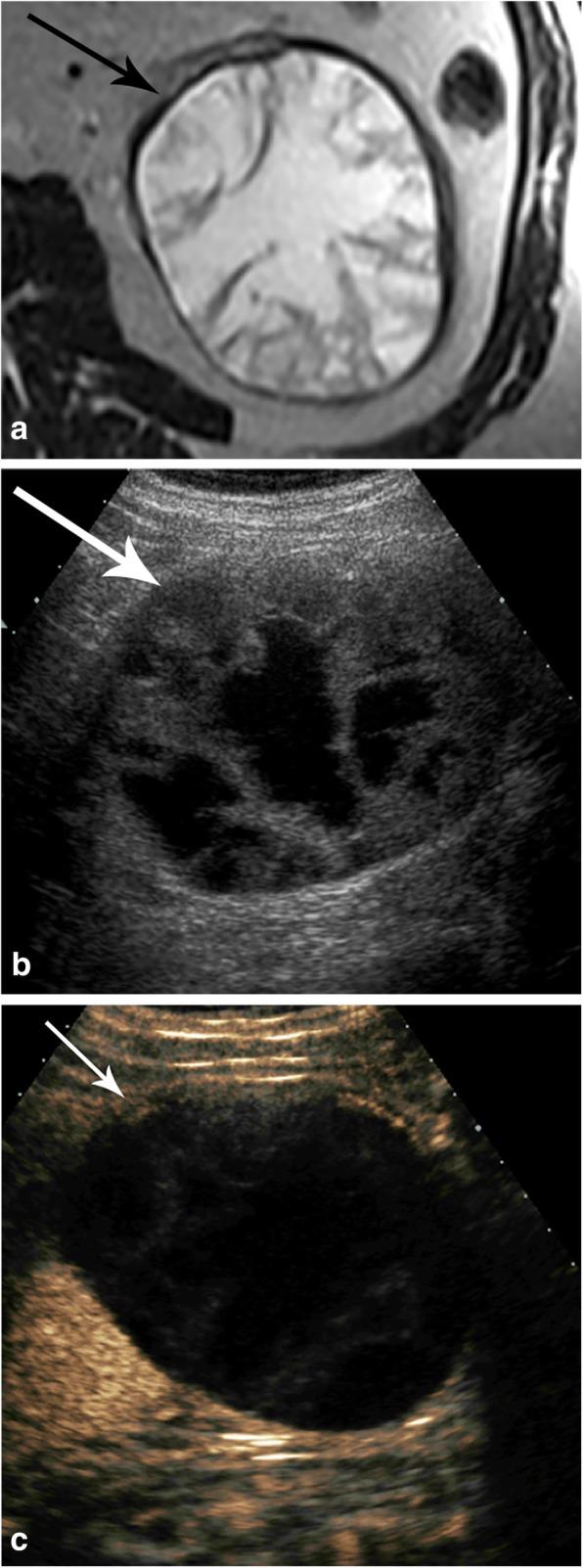
Image suspected of being a complex hemorrhagic cyst larger than 3 cm in size, which was classified as Bosniak IIF, and then later reclassified as a Bosniak II cyst by contrast-enhanced US. **a** Axial T2 MR showing a complex renal cyst with thick septa (arrow). **b** Unenhanced US of the same lesion showing cystic component and echogenic material within the cyst (arrow). **c** Contrast-enhanced US showing the absence of enhancement of septa (arrow). The lesion was reclassified as a Bosniak I simple cyst not requiring follow-up

Historically, a percutaneous biopsy was not recommended in complex renal cysts because of the high rates of false-negative results, due to cyst septations; however, recent studies have described a better diagnostic yield [31, 58–61]. Nodular areas found inside the cysts should always be included in the RTB (Fig. 20).

## Multifocal small renal masses and active surveillance

It is important to clarify the meaning of multifocality. It is defined as the presence of more than one tumor, which can be unilateral or bilateral in SRMs. Multifocal SRMs can be synchronic or metachronous and are considered as synchronous if appearing within less than 6 months [62, 63].

It is more difficult to establish AS protocols for patients with multifocal SRMs than for patients with solitary SRMs. Indeed, there is no consensus in this regard in urological guidelines, and there is scarce literature about the clinical behavior of these masses, with only short series having been evaluated [63–66]. However, there is a real increase in the prevalence of multifocal renal tumors [67].

The presence of multifocal renal tumors is not unusual, and according to histological series after nephrectomy, they can appear in approximately 25% of patients with RCC [68, 69]. Bilateral renal tumors have been observed in 90% of patients with multifocal tumors. The most frequent histological RCC variant associated with a multifocality is the papillary subtype, but any histologic RCC can be multifocal [62]. Multifocal renal tumors can be sporadic or associated with genetic syndromes. In patients with multifocal sporadic tumors, the recommendation of AS is the same as in single tumors. The linear GR of all the SRMs is measured and calculated in routine clinical practice.

The management of multifocal renal tumors with genetic predisposition is even more challenging, and at present, there is no urological or oncological consensus in this regard. Approximately 5 to 8% of renal cancers have a hereditary component, and some studies have reported that in up to 58% of these tumors, family genetics plays a significant role [70].

**Fig. 21:**
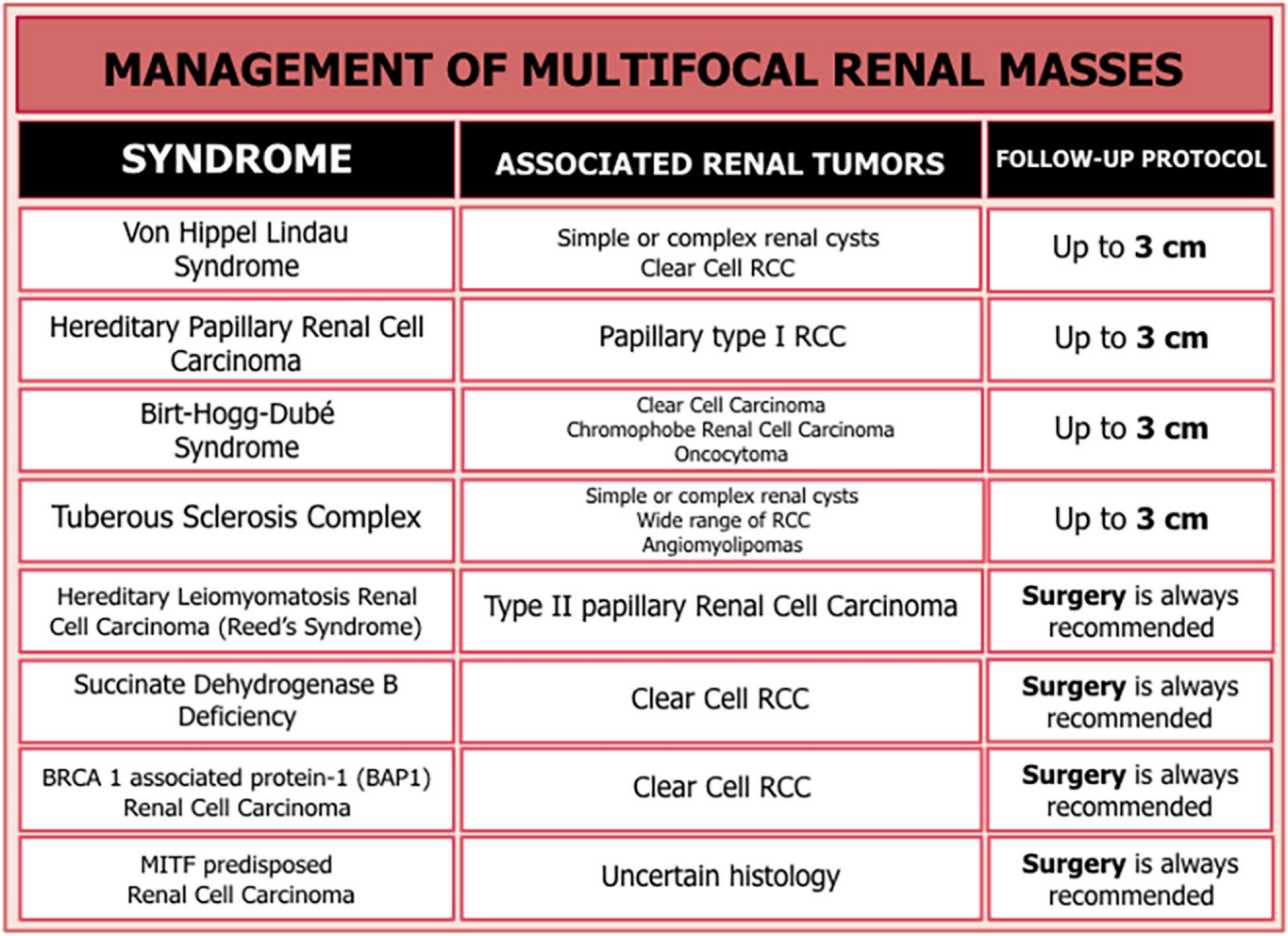
Multifocal small renal masses (SRM), genetic syndromes classification, type of associated renal tumors, and suggested follow-up by active surveillance (AS)

In patients with hereditary syndromes, RCC appears at a younger age and have a greater propensity to develop synchronic and metachronous renal tumors. As a result, the likelihood of these patients requiring multiple surgical kidney procedures should be taken into account [71]. It is important to diagnose these syndromes early. The most frequent syndromes associated with multiple renal tumors are Von Hippel-Lindau (VHL), hereditary papillary renal cell carcinoma (HPRC), tuberous sclerosis complex (TSC), hereditary leiomyomatosis RCC (HLRCC), succinate dehydrogenase B deficiency (SDHB), and Cowden syndromes. The more recently described syndromes are associated with genetic alterations in BRCA1-associated protein-1 (BAP1) or microphthalmia-associated transcription factor (MITF). In patients with multifocal sporadic or hereditary SRMs, RTB is always recommended, if technically possible [63]. The ideal follow-up includes size measurement and linear GR calculation of all tumors, but this can be technically challenging and time-consuming. In the presence of multiple multifocal renal masses, we recommend measuring only SMRs larger than 2 cm or those that seem to have more rapid growth (Fig. 21).

In patients with multifocal SRMs, the main objectives of management are preserving renal function, avoiding surgical morbimortality, achieving optimal oncological control, and providing long-term cancer recurrence-free survival. AS is the treatment of choice in sporadic multifocal and some hereditary SRMs. In classical hereditary syndromes (VHL, HPRC, and BHD), AS is recommended until the tumor reaches 3 cm. In HLRCC and SDHB, the risk of metastatic disease in smaller tumors is high. Therefore, surgery is always recommended. There is no consensus on new syndromes, and therefore, immediate surgery after diagnosis is also recommended, even in very small tumors [72, 73].

## Conclusion

AS is effective in the treatment of SRMs. It is mandatory for radiologists to know how to most adequately manage AS in SRMs in order to achieve the most optimal results. Complicated renal cysts and multifocal renal masses need a specific approach.

## Data Availability

Not applicable

## Abbreviations

AS: Active surveillance
ASCO: American Society of Clinical Oncology
AUA: American Urology Association
CEUS: Contrast-enhanced ultrasound
GR: Growth rate
HLRCC: Hereditary leiomyomatosis renal cell carcinoma
HPRC: Hereditary papillary renal cell carcinoma
NCCN: National Comprehensive Cancer Network
RCC: Renal cell carcinoma
RTB: Renal tumor biopsy
SDHB: Succinate dehydrogenase B
SRM: Small renal mass
TSC: Tuberous sclerosis complex
TSTC: Too small to be characterized renal masses
VHL: Von Hippel-Lindau
WW: Watchful waiting
